# Juvenile Granulosa Cell Tumor in an Adolescent With Xeroderma Pigmentosum: A Rare Case and Implications for Surveillance

**DOI:** 10.7759/cureus.91331

**Published:** 2025-08-31

**Authors:** Islam Erraoui, Ayad Ghanam, Manal Azizi, Houssain Benhaddou, Maria Rkain

**Affiliations:** 1 Pediatrics, Mohammed VI University Hospital Center, Oujda, MAR; 2 Medicine and Pharmacy, Mohammed First University, Oujda, MAR; 3 Pediatric Gastroenterology, Mohammed VI University Hospital Center, Oujda, MAR

**Keywords:** adolescents, dna repair deficiency, gynecological surveillance, juvenile granulosa cell tumor, ovarian tumor, xeroderma pigmentosum

## Abstract

We report the case of a 14-year-old girl with xeroderma pigmentosum who was incidentally found to have a juvenile granulosa cell tumor of the ovary during routine imaging. The tumor was surgically removed, and no signs of hormonal activity or metastasis were observed. The patient had an uneventful recovery and required no additional therapy. This case underscores a rare but noteworthy association between a DNA repair disorder and early-onset gynecological tumors, highlighting the importance of regular gynecological surveillance in affected patients. Early detection may improve clinical outcomes and reduce the need for aggressive treatment.

## Introduction

Juvenile granulosa cell tumors (JGCTs) are rare ovarian neoplasms, accounting for approximately 5% of granulosa cell tumors and 2%-5% of all ovarian cancers [1]. They typically occur in girls under 20 years of age, with a median age at diagnosis between seven and eight years. Clinically, they most often present with isosexual precocious puberty, menstrual irregularities, or a pelvic mass detected on imaging [2].

The standard treatment involves fertility-sparing surgery, typically unilateral adnexectomy, sometimes followed by chemotherapy in advanced or aggressive disease. The prognosis is generally favorable in early-stage disease (International Federation of Gynecology and Obstetrics (FIGO) stages IA-IB), with a five-year survival rate of 90%-100% [3]. Xeroderma pigmentosum (XP) is a rare genetic disorder characterized by a defect in DNA repair following ultraviolet (UV) exposure, resulting in a high predisposition to skin cancers [4]. Although internal tumors are uncommon, recent reports describe gynecological tumors, including ovarian JGCT, in adolescents with XP [5].

We report the case of a 14-year-old girl with xeroderma pigmentosum who developed a juvenile granulosa cell tumor of the ovary, incidentally discovered without typical endocrine manifestations. This rare case highlights the importance of enhanced onco-gynecological surveillance in XP patients and contributes to the limited literature on this association.

## Case presentation

**Figure 1:**
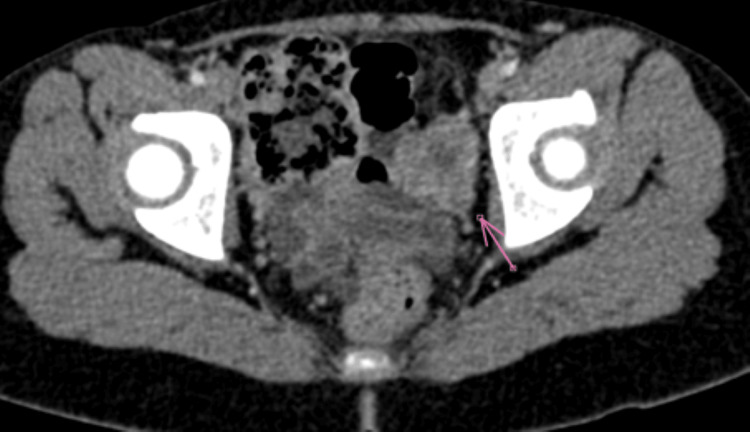
Abdominopelvic CT showing a suspicious left lateral uterine mass (arrow) of likely ovarian origin.

We report the case of a 14-year-old adolescent born to non-consanguineous parents, followed since early childhood for xeroderma pigmentosum, a rare genodermatosis characterized by extreme sensitivity to UV radiation and a marked predisposition to cutaneous cancers. Her medical history included multiple surgeries for cutaneous squamous and basal cell carcinomas, as well as surgery for a toe myeloma (Figure 1).

The patient was admitted for routine dermatological surveillance, during which a suspicious ovarian mass was incidentally detected on abdominal ultrasound as part of her follow-up. It appeared predominantly solid and homogeneous with a few central anechoic areas, but its precise characteristics were difficult to determine due to its size and location.

Upon admission, the patient was conscious, afebrile, and hemodynamically stable. Physical examination revealed multiple hyperpigmented and hypopigmented macules and small papules on the face and photo-exposed areas, along with scattered nodules in some regions, consistent with her underlying condition. The lesions were predominantly distributed over the face, neck, forearms, and dorsal aspects of the hands. There was no involvement of the mucous membranes. Abdominal examination showed a soft, non-distended abdomen without palpable mass or guarding; hernial orifices were clear, and bowel sounds were normal. No other abnormalities were noted on clinical examination.

**Figure 2:**
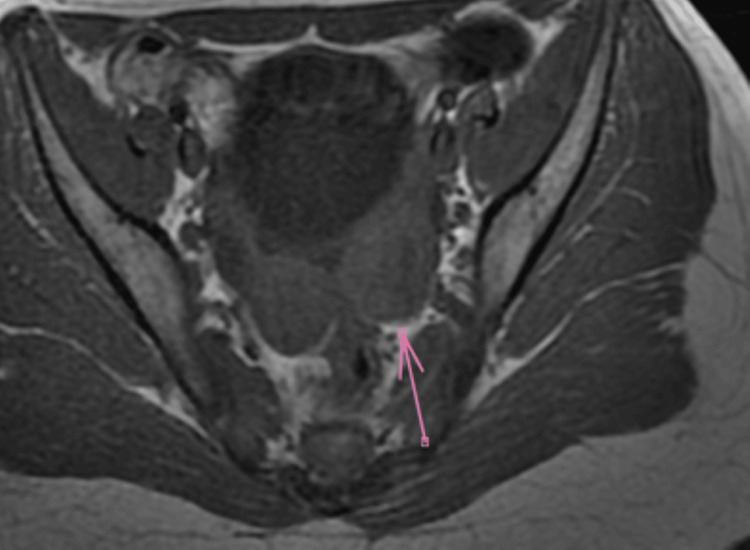
Axial pelvic MRI in T1-weighted sequence showing the presence of a mass classified as O-RADS 4 (arrow), highly suspicious for malignancy. O-RADS: Ovarian-Adnexal Reporting and Data System.

Biological tests showed normal complete blood count, hepatic and renal function, and no signs of tumor lysis. A pelvic CT scan demonstrated a left latero-uterine soft tissue mass, apparently arising from the ovary on ultrasound correlation, with irregular but fairly well-defined contours, showing heterogeneous enhancement after contrast injection, measuring 33 × 27 mm. Bilateral inguinal lymph nodes are subcentimetric (Figure 2).

**Figure 3:**
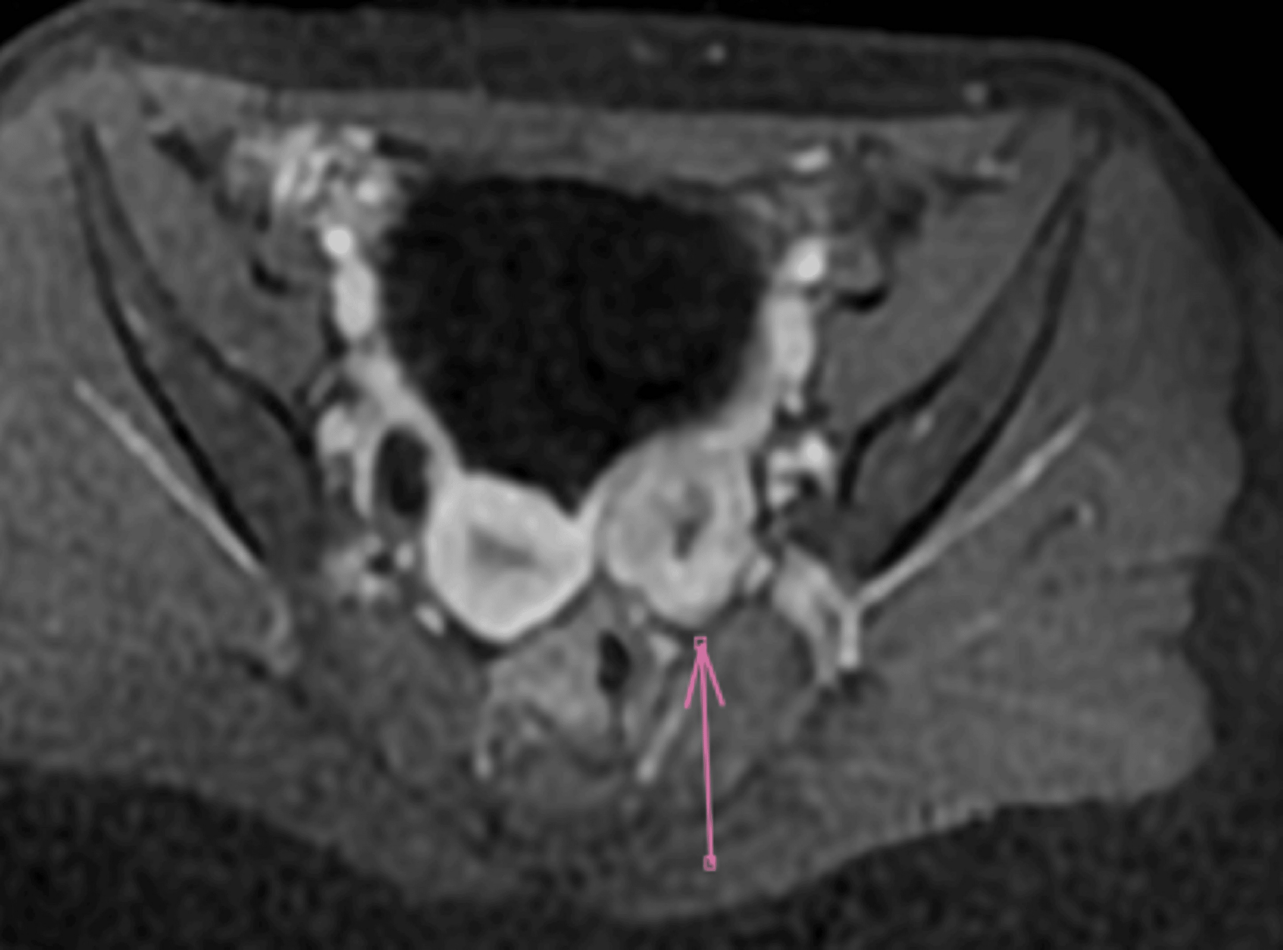
Axial pelvic MRI in T2-weighted imaging after gadolinium injection showing the presence of a mass classified as O-RADS 4 (arrow), highly suspicious for malignancy.

**Figure 4:**
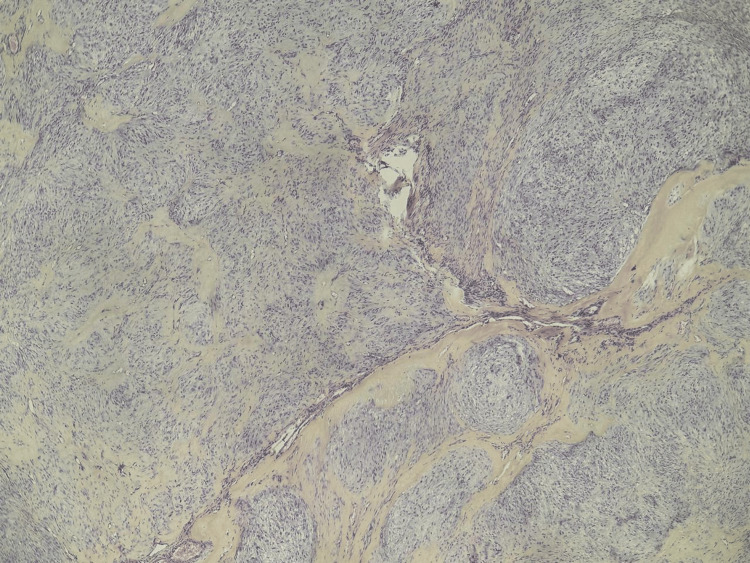
Histological section (H&E, ×4) showing a solid nodular tumor of monomorphic oval-to-spindle cells with grooved (“coffee-bean”) nuclei, rare mitoses, and fibrous stroma.

Pelvic MRI demonstrates a left latero-uterine mass with irregular contours, intermediate T2 and isointense T1 signals, restricted diffusion with apparent diffusion coefficient (ADC) drop, and type 1 gadolinium enhancement, containing a central area of necrosis. The lesion measures 31 × 21 mm, abutting the uterus medially while sparing the internal iliac pedicle laterally. The left ovary is not clearly visualized; the right ovary measures 12 × 12 mm with follicular appearance. The uterus is anteflexed and retroverted, measuring 59 × 22 mm, with normal signal and morphology. Cervical signal and thickness, as well as the parametrium, are preserved. No bowel wall thickening is seen. External iliac lymph nodes are subcentimetric. Minimal intrapelvic fluid is present (Figures 3, 4).

Serum tumor markers were all within normal limits: inhibin B 80 pg/mL, α-fetoprotein (AFP) 5 ng/mL, β-human chorionic gonadotropin (β-hCG) 2 mIU/mL, cancer antigen 125 (CA-125) 20 U/mL, and lactate dehydrogenase (LDH) 180 U/L. All values were therefore considered negative. An exploratory laparoscopy followed by left adnexectomy was performed by the pediatric surgery team. Intraoperatively, the left ovarian mass measured 3.5 × 3.5 × 1 cm was well-circumscribed and predominantly solid, with no evidence of capsular invasion. The contralateral ovary and uterus appeared normal, and no ascites, peritoneal implants, or abnormal lymph nodes were noted.

**Figure 5:**
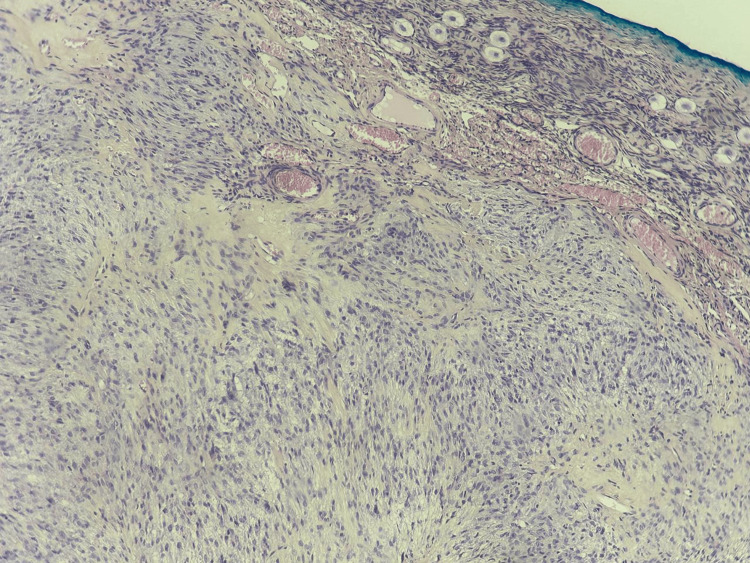
Histological section (H&E, ×10) showing a solid nodular tumor of monomorphic oval-to-spindle cells with grooved (“coffee-bean”) nuclei, rare mitoses, and fibrous stroma.

Histological examination of the lesion revealed a predominantly solid tumor proliferation, composed of nodules separated by fibrous septa. The proliferation consisted of monotonous oval to occasionally spindle-shaped cells with moderately abundant cytoplasm, which was sometimes eosinophilic and at other times luteinized and vacuolated. The nuclei were round with fine chromatin, showing occasional nuclear grooves giving a “coffee bean” appearance in some areas. Mitotic figures were estimated at 2 per 10 high-power fields (HPF). The stroma was fibrous. No Call-Exner bodies were identified (Figure 5).

**Figure 6:**
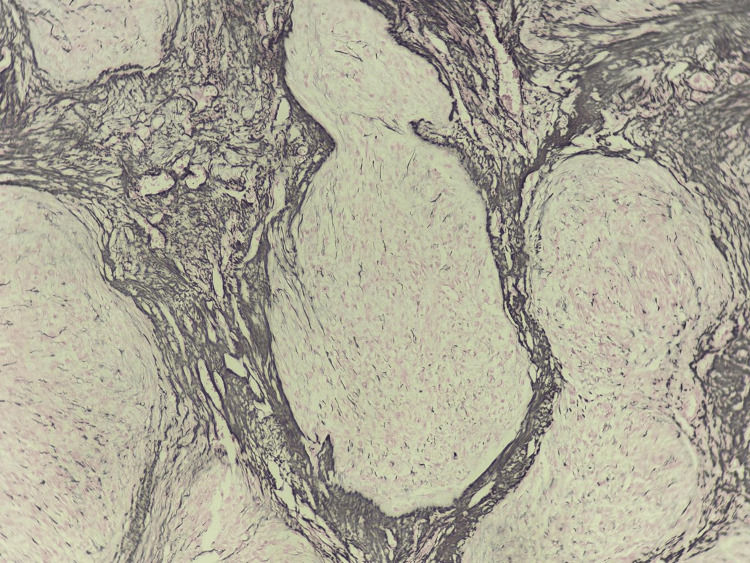
Immunohistochemistry: reticulin stain positive, highlighting the boundaries of tumor nodules.

**Figure 7:**
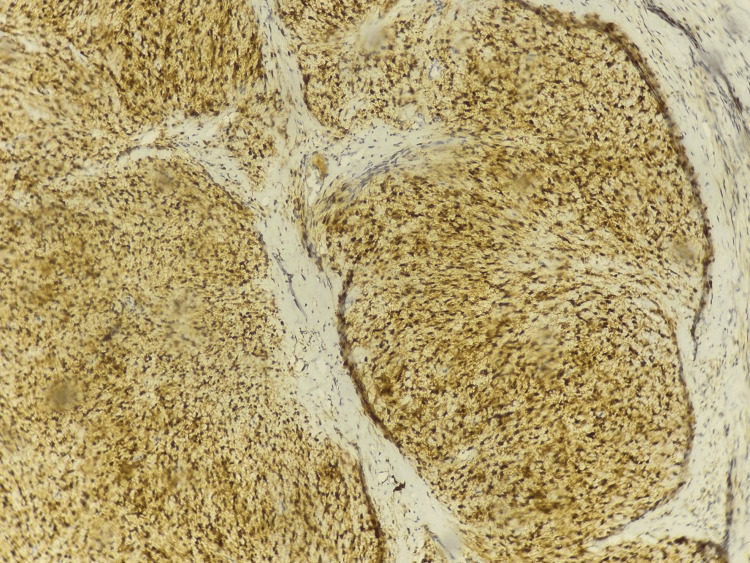
Immunohistochemistry showing positive tumor cell staining for inhibin.

**Figure 8:**
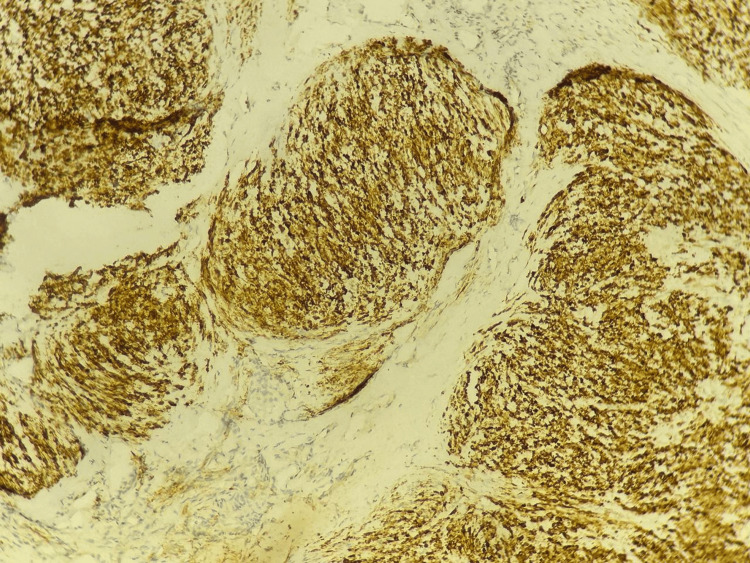
Immunohistochemistry showing positive tumor cell staining for calretinin.

An immunohistochemical study was performed, showing positive staining of the tumor cells for inhibin and calretinin and absence of staining for GATA-binding protein 3 (GATA3). A special reticulin stain was also performed, which was positive, delineating the tumor nodules. These histological and immunohistochemical features are compatible with a juvenile granulosa cell tumor (Figures 6-8).

The tumor was confined to the left ovary without capsule rupture or evidence of metastasis, corresponding to FIGO stage IA. Postoperative recovery was uneventful. The patient underwent regular clinical and radiological surveillance, including chest X-rays and abdominopelvic ultrasounds. Follow-up showed no recurrence. A multidisciplinary team concluded that neither chemotherapy nor radiotherapy was required.

## Discussion

The association between a juvenile granulosa cell tumor and xeroderma pigmentosum represents an exceptional clinical scenario, illustrating the oncogenic potential of congenital DNA repair defects [5]. These tumors often present with signs of hyperestrogenism, such as isosexual precocious puberty, menstrual irregularities, or a pelvic mass [6]. In XP patients, cutaneous malignancies are the most frequently reported due to hypersensitivity to UV radiation resulting from defective nucleotide excision repair (NER) of single-strand DNA breaks [7]. However, certain clinical subtypes, especially XP type C, carry an increased risk of internal malignancies [8].

A retrospective Tunisian study reported five cases of gynecological cancers in xeroderma pigmentosum, complementation group C (XP-C) patients carrying the V548A fs X572 mutation, including uterine sarcomas, a uterine leiomyosarcoma, and an ovarian adenocarcinoma, with a median age of 22.4 years [5]. These findings suggest that the DNA repair defect in XP-C may promote gynecological tumorigenesis independently of cutaneous cancers [5].

In a multicenter review by Bradford et al. (2011) involving 106 XP patients, a significantly increased risk of internal tumors, including sarcomas, carcinomas, and CNS tumors, was observed in XP-C patients, suggesting that NER deficiency may affect tissues beyond the skin [9]. Similarly, Kraemer et al. (2003) reported that XP-C patients develop deep-seated tumors at younger ages compared to the general population, with a nearly 10-fold higher rate of internal cancers [10].

Our case aligns with the findings of Yurchenko et al. (2023), who described a series of early-onset gynecological tumors, including a JGCT, in XP adolescents, highlighting the likely role of NER deficiency in ovarian tumorigenesis [11]. Moreover, international cohort analyses have shown that gynecological tumors account for 13% of internal malignancies in XP patients, with a particularly high risk among carriers of the delTG mutation in the *XPC* gene, warranting regular gynecological screening from adolescence onward [8].

In a U.S. pediatric study, two XP-C adolescents aged 15 and 17 developed gynecological tumors, including a possible granulosa-type ovarian tumor, suggesting that DNA repair defects (NER pathway) may predispose to the early onset of these malignancies [12]. These findings underscore the importance of systematic imaging surveillance, as in our case, where the JGCT was incidentally detected, enabling early diagnosis of these rare but serious tumors [9].

## Conclusions

This case report sheds new light on the emerging association between xeroderma pigmentosum type C and early-onset gynecological neoplasms, particularly juvenile granulosa cell tumors. The development of an ovarian tumor in the context of a congenital defect in DNA repair mechanisms supports the hypothesis of a broader systemic oncogenic predisposition in patients with XP, extending beyond the well-characterized cutaneous manifestations. These findings underscore the imperative for proactive and prolonged gynecological surveillance in affected adolescents. They also call for increased clinician awareness regarding the comprehensive management of patients with XP, including multidisciplinary follow-up to anticipate and address extra-cutaneous tumor complications.
